# Biological substrates of migraine

**DOI:** 10.1186/1129-2377-16-S1-A19

**Published:** 2015-09-28

**Authors:** Paolo Calabresi

**Affiliations:** Clinica Neurologica, Dipartimento Medicina, Università degli Studi di Peugia, Perugia, Italy

Migraine episodes are probably originated by central mechanisms in brain areas able to cause the classical neurological symptoms of prodromes and aura. Conversely, the headache phase begins with the activation of meningeal nociceptors at the origin of the trigeminovascular system. Recent information has postulated that the occurrence of aura can activate nociceptors in the meninges. Conversely, the mechanisms by which common prodromes initiate the headache phase or what sequence of events triggers activation of the meningeal nociceptors is not yet understood. However, a mechanistic analysis for a common factor in migraine clinical features strongly suggests a genetic predisposition to generalized neuronal hyperexcitability.

Hypothalamic neurons are sensitive to changes in physiological and emotional homeostasis and they might activate meningeal nociceptors by altering the balance between parasympathetic and sympathetic influence in the meninges. Accordingly, hypothalamic neurons that contain dopamine, histamine, and orexin, and brainstem neurons that contain noradrenaline and serotonin send inputs to trigemino-thalamic neurons in sensory thalamic nuclei. These neurotransmitters can shift the activity of thalamic neurons.

Clinical and preclinical studies suggest that migraine aura is caused by cortical spreading depression (CSD), a slowly propagating wave of depolarization/excitation followed by inhibition in cortical neurons and glia. In the cortex, the initial membrane depolarization is associated with a large efflux of potassium, influx of sodium and calcium and release of glutamate. Interestingly, endogenous CGRP is released in the cortical tissue during CSD, and CGRP receptor antagonists have an inhibitory effect on CSD, suggesting a critical role of CGRP in this phenomenon. The demonstration that CGRP antagonism reduces CSD supports the possible use of drugs targeting central CGRP receptors as antimigraine agents.

Finally, among the various pathophysiological conditions controlling the expression and the features of headache in migraine, medication-overuse headache (MOH) plays a central role. MOH is a clinically important entity and it is now well documented that the regular use of acute symptomatic medication by people with migraine increases the risk of aggravation of the primary headache. MOH is one of the most common causes of chronic migraine-like syndrome. We have analyzed the possible mechanisms underlying sensitization in MOH by comparing these mechanisms with those reported for other forms of drug addiction. Recent data support the evidence for cognitive impulsivity in drug overuse in headache and in other forms of addiction associated with dysfunction of the frontostriatal system and an integrative hypothesis for compulsive reward-seeking in MOH.

